# Difficult biliary cannulation during ERCP: techniques and an evidence-informed practical algorithm

**DOI:** 10.3389/fmed.2026.1924528

**Published:** 2026-09-28

**Authors:** Haichuan Zhou, Shengjie Yang, Hongyu Li

**Affiliations:** Department of Gastroenterology, Changxing County People's Hospital, Huzhou, Zhejiang, China

**Keywords:** difficult biliary cannulation, ERCP, EUS-guided biliary drainage, needle-knife fistulotomy, post-ERCP pancreatitis, precut sphincterotomy, transpancreatic sphincterotomy

## Abstract

Difficult biliary cannulation remains the pivotal technical barrier in therapeutic endoscopic retrograde cholangiopancreatography (ERCP) and is a major modifiable driver of post-ERCP pancreatitis (PEP). Contemporary practice has moved from prolonged standard attempts toward earlier recognition of difficult access, guidewire-based primary cannulation, targeted rescue techniques, and routine pancreatitis prophylaxis. This review summarizes current evidence and proposes a practical, evidence-informed algorithm for native papilla ERCP. Difficult cannulation is best recognized using objective procedural thresholds, including more than five papillary contacts, more than 5 min after papillary visualization, or more than one unintended pancreatic duct (PD) cannulation or opacification. Primary cannulation should use a wire-guided approach with careful control of papillary trauma and contrast injection. Once difficulty is declared, rescue technique selection should follow procedural anatomy and clinical context rather than a rigid sequence: repeated PD access favors double-guidewire (DGW), transpancreatic biliary sphincterotomy (TPBS), or PD stent-assisted approaches; a bulging or favorably oriented papilla favors early needle-knife fistulotomy (NKF); and failed access despite endoscopist expertise should prompt escalation rather than continued trauma. Rectal nonsteroidal anti-inflammatory drugs should be administered before or during ERCP in patients without contraindications; prophylactic PD stenting should be used after repeated or deep PD access and according to the rescue technique and overall PEP risk, with appropriate hydration in suitable patients. Local postprocedural cryoprevention with papillary ice-water irrigation is an emerging adjunct supported by one multicenter randomized trial, although its incremental benefit alongside standard prophylaxis remains uncertain. When ERCP fails, repeat expert ERCP, endoscopic ultrasound-guided rendezvous (EUS-RV) or biliary drainage (EUS-BD), and percutaneous transhepatic biliary drainage (PTBD) should be selected according to indication, urgency, anatomy, patient stability, and local expertise. The algorithm distinguishes guideline-supported decision nodes from areas in which comparative evidence is limited and sequencing represents a practical expert synthesis.

## Introduction

Endoscopic retrograde cholangiopancreatography (ERCP) has evolved from a diagnostic test to a predominantly therapeutic intervention for choledocholithiasis, biliary strictures, cholangitis, bile leaks, and selected pancreaticobiliary disorders. Selective biliary cannulation is the gateway step for nearly all therapeutic ERCP, yet it is also the point at which mechanical trauma, papillary edema, thermal injury, hydrostatic injury, and repeated pancreatic duct manipulation may initiate post-ERCP pancreatitis (PEP). For surgeons and endoscopists managing biliary disease, difficult biliary cannulation is therefore more than a technical inconvenience: it is a procedural inflection point that determines whether the case proceeds safely, requires advanced access, or should be converted to another drainage strategy.

The modern approach is defined by two principles. First, standard cannulation should not be continued indefinitely once objective markers of difficulty appear. Second, rescue should be matched to the anatomy encountered at the papilla rather than applied as a fixed sequence. Current guidelines and meta-analyses support wire-guided primary cannulation, early use of advanced techniques by trained operators, and aggressive prevention of PEP with rectal nonsteroidal anti-inflammatory drugs (NSAIDs), selective PD stenting, and judicious intravenous hydration (1–6). This review provides a concise evidence-based synthesis and a practical algorithm for difficult biliary cannulation in patients with a native papilla and conventional anatomy, with comments on altered anatomy and failed ERCP bailout. The proposed algorithm is evidence-informed rather than a validated fixed sequence. Guideline-supported components are identified according to recommendation strength and evidence quality where available, whereas the precise sequencing among rescue techniques is presented as a practical synthesis that should be individualized according to papillary morphology, PD access, ERCP indication, underlying anatomy, previous interventions, patient-related PEP and bleeding risk, and operator and institutional expertise.

## Definition and early risk stratification

The European Society of Gastrointestinal Endoscopy (ESGE) definition remains clinically useful because it forces early recognition. Difficult biliary cannulation is present when any of the following occurs: more than five contacts with the papilla, more than 5 min spent attempting cannulation after visualization of the papilla, or more than one unintended pancreatic duct cannulation or opacification (2). This “5-5-1” concept is easy to remember and should be part of the verbal time-out during ERCP. It is imperfect, because a single traumatic passage into the pancreatic duct in a young patient with normal bilirubin may be more consequential than several gentle attempts in an elderly patient with a dilated bile duct. Nonetheless, using a threshold prevents the common error of continuing standard attempts until edema and bleeding have already narrowed the therapeutic window.

Risk stratification begins before the endoscope reaches the papilla. Patient factors associated with PEP include suspected sphincter of Oddi disorder, prior PEP, female sex, younger age, normal serum bilirubin, and nondilated ducts; procedure-related factors include difficult cannulation, pancreatic injection, pancreatic guidewire passages, precut sphincterotomy in some settings, and papillectomy (3, 4). Papillary morphology adds another layer. The Haraldsson classification describes regular, small, protruding or pendulous, and creased or ridged papillae (7). A 2024 systematic review found that difficult cannulation was lowest in regular papillae and highest in creased or ridged papillae, while PEP was highest in small papillae (8). These observations support tailoring the rescue strategy to visible anatomy rather than waiting for failure. A 2025 secondary analysis of the Stent versus Indomethacin trial dataset further showed that difficult cannulation confers the greatest pancreatitis risk when it occurs in patients with high baseline PEP risk, supporting early rescue and prophylaxis rather than prolonged attempts (9). Representative endoscopic images of the four Haraldsson papillary types are shown in Figure 1 (10).

**Figure 1:**
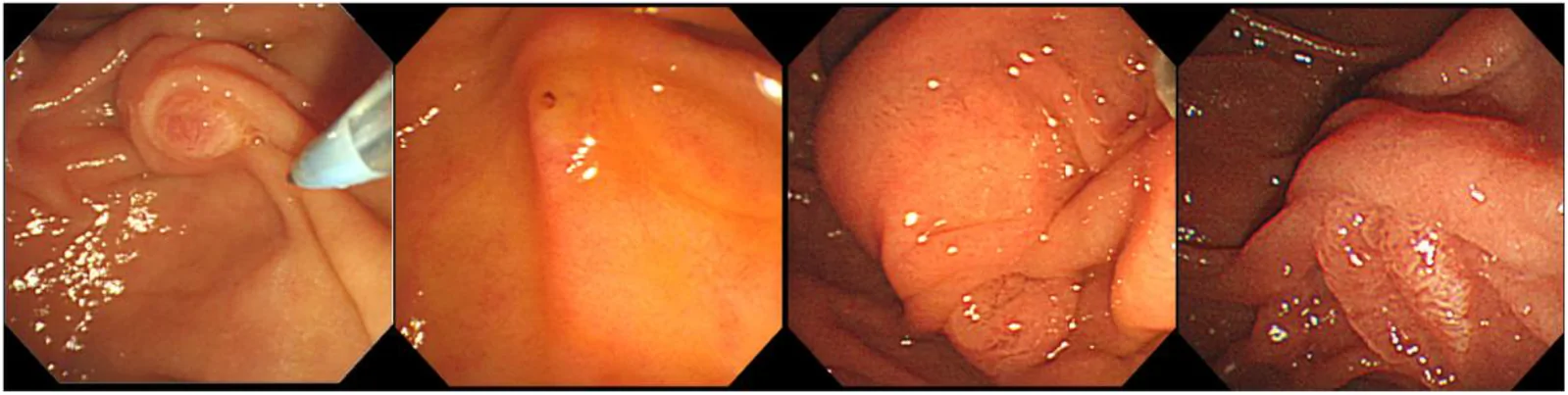
Representative endoscopic appearances of the haraldsson classification, from left to right: type 1, regular papilla; type 2, small or flat papilla (diameter <=3 mm); type 3, protruding or pendulous papilla; and type 4, creased or ridged papilla. Reproduced without modification from Chen et al. (10) under the Creative Commons Attribution 4.0 International License (CC BY 4.0).

## Interpreting the proposed algorithm

The algorithm should be read as a flexible decision framework, not a mandatory ladder. Several core nodes are directly supported by guidelines: the ESGE 5-5-1 definition of difficult cannulation is a weak recommendation based on low-quality evidence; wire-guided primary cannulation is strongly recommended by ESGE and WEO on moderate-quality evidence and conditionally suggested by ASGE on moderate-quality evidence; rectal NSAIDs are strongly recommended by ASGE and WEO on moderate-quality evidence; and prophylactic PD stenting is strongly recommended by ASGE after repeated or deep PD access and by ESGE after pancreatic guidewire-assisted cannulation, with additional guideline support after TPBS (1, 2, 4, 6). In contrast, no high-quality evidence establishes a universal order among DGW, TPBS, PD stent-assisted rescue, and precut techniques. Comparative randomized trials and network meta-analyses inform selected pairwise choices, but the sequence shown in Figures 2, 3 represents a practical synthesis and should be adapted to papillary morphology, whether PD access is already present, ERCP indication and urgency, anatomy, prior intervention, PEP and bleeding risk, and operator expertise (1, 11–18).

**Figure 2:**
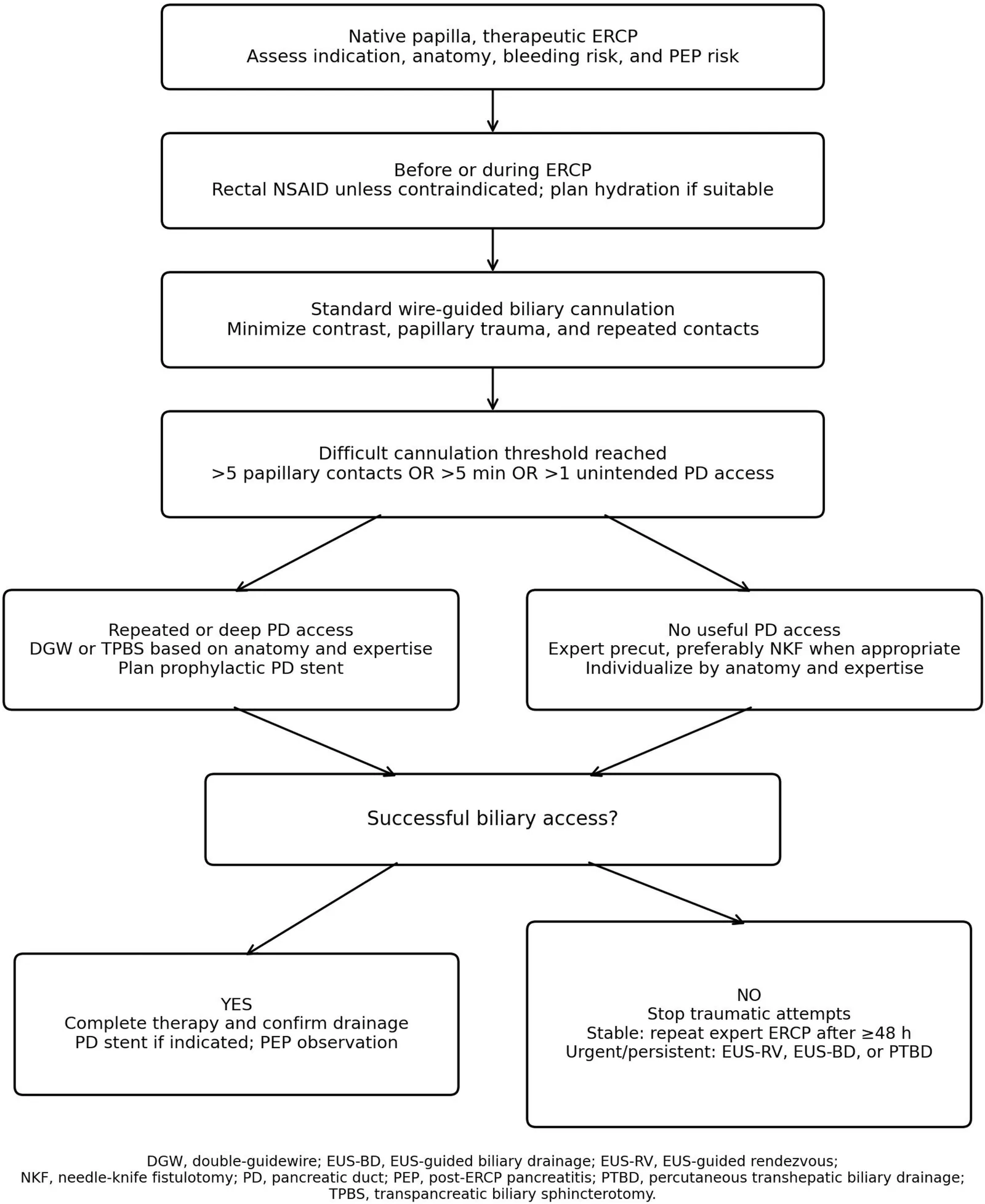
Proposed evidence-informed, non-rigid algorithm for native papilla ERCP. PEP prophylaxis begins before or during ERCP and is reassessed when the PD is repeatedly or deeply instrumented. Rescue technique selection is anatomy- and context-directed rather than a mandatory sequence. DGW, double-guidewire; EUS-BD, EUS-guided biliary drainage; EUS-RV, EUS-guided rendezvous; NKF, needle-knife fistulotomy; PD, pancreatic duct; PEP, post-ERCP pancreatitis; PTBD, percutaneous transhepatic biliary drainage; TPBS, transpancreatic biliary sphincterotomy.

**Figure 3:**
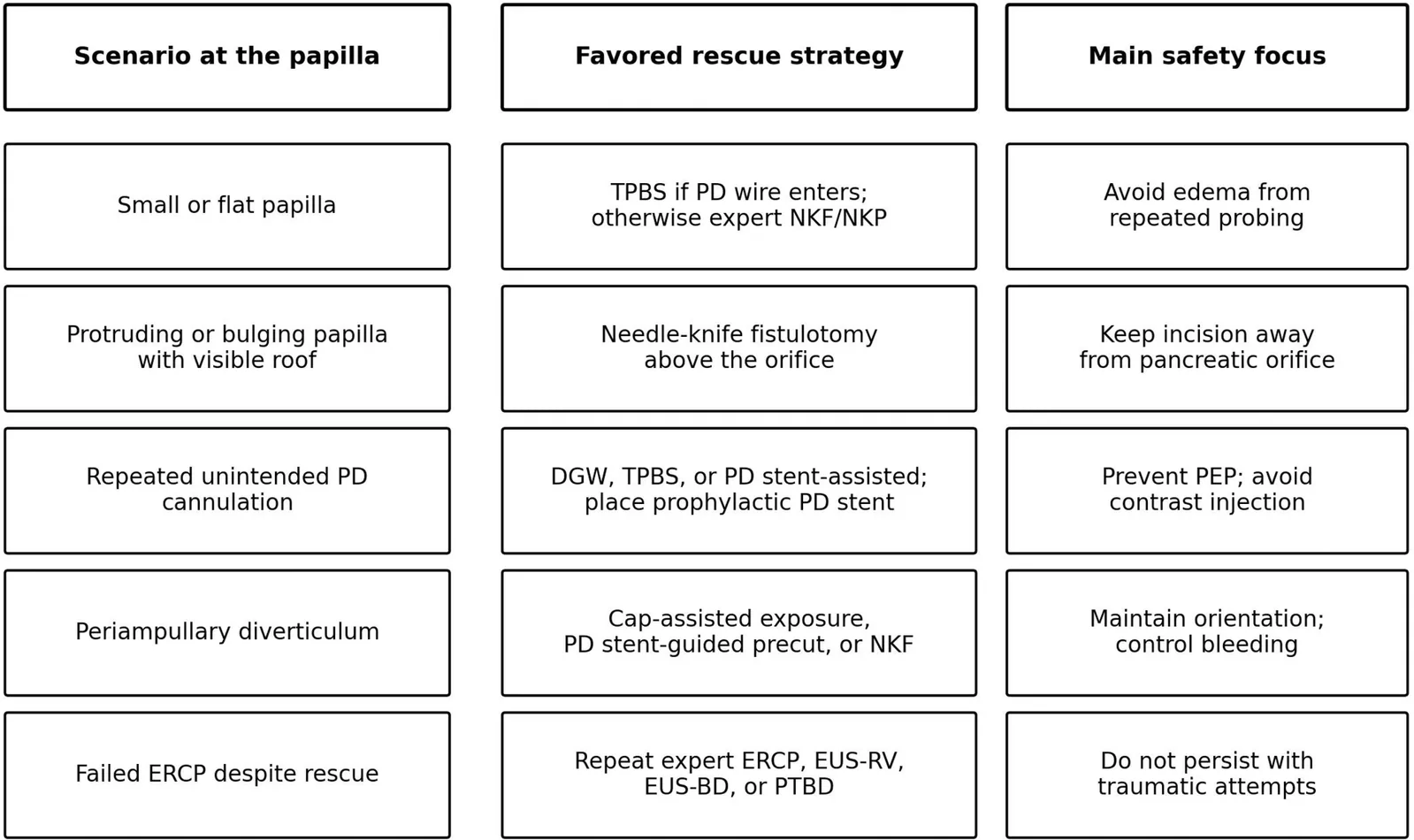
Rescue technique selection should follow papillary morphology, PD access, clinical indication, and availability of expertise. The matrix is a cognitive aid rather than a priority ranking and should be adapted to patient stability, ductal dilation, antithrombotic status, previous interventions, and local resources. DGW, double-guidewire; EUS-BD, EUS-guided biliary drainage; EUS-RV, EUS-guided rendezvous; NKF, needle-knife fistulotomy; NKP, needle-knife papillotomy; PD, pancreatic duct; PTBD, percutaneous transhepatic biliary drainage; TPBS, transpancreatic biliary sphincterotomy.

## Primary cannulation: controlled wire-guided access

Wire-guided cannulation should be the default primary technique. In contrast-assisted cannulation, repeated injections at the papillary orifice can inadvertently opacify the PD and increase hydrostatic injury. Wire-guided access uses a hydrophilic guidewire to test the ductal axis with minimal contrast. A 2022 Cochrane review concluded that guidewire-assisted cannulation probably reduces PEP compared with contrast-assisted cannulation and may increase primary cannulation success (5). ESGE and WEO recommend wire-guided primary cannulation with strong recommendations based on moderate-quality evidence, while ASGE suggests wire-guided over contrast-guided cannulation with a conditional recommendation based on moderate-quality evidence (1, 2, 4). Advanced cannulation techniques for difficult biliary access are summarized in Table 1.

**Table 1:** Advanced cannulation techniques for difficult biliary access.

Technique	Best-fit scenario	Key advantages	Important limitations/precautions
Wire-guided standard cannulation	First-line approach in native papilla ERCP	Reduces contrast injection; probably lowers PEP risk; widely available	Avoid repeated papillary trauma; declare difficulty early
Double-guidewire (DGW) technique	Repeated unintended pancreatic duct access with stable wire position	Uses pancreatic wire to straighten channel and redirect second wire	Pancreatic manipulation may increase PEP risk; prophylactic PD stenting should be planned when feasible after PGW/DGW-assisted cannulation (1, 2, 4).
Transpancreatic biliary sphincterotomy (TPBS)	Small or flat papilla; use only when stable PD guidewire access is already established	High rescue cannulation success in selected RCTs	Requires septal incision; prophylactic PD stenting should generally be planned when feasible (1, 2).
PD stent-assisted cannulation/precut	PD access already achieved with need to protect drainage or guide incision	Stabilizes papilla and maintains pancreatic drainage	Stent follow-up is needed if retained; useful when PD access is already available.
Needle-knife fistulotomy (NKF)	Bulging/protruding papilla, visible roof, duct dilated to papilla; may also be considered for a selected small/flat papilla without useful PD access when an adequate roof is present	Avoids pancreatic orifice; useful as early expert rescue	Requires expertise and favorable anatomy; routine intentional PD stenting is not universally required when the PD has not been instrumented.
Needle-knife papillotomy (NKP)	Visible orifice but deep biliary access fails	Direct route from papillary orifice toward bile duct	Closer to pancreatic orifice; careful incision control required
EUS-RV/EUS-BD/PTBD	Failed ERCP or inaccessible papilla	Provides rescue drainage without persistent papillary trauma	Choice depends on benign vs. malignant disease, urgency, anatomy, patient stability, future surgical planning, and local expertise.

Technique matters as much as device choice. The papilla should be placed in a stable, en face position when possible, with the sphincterotome bowed toward the expected 11 to 12 o'clock biliar*y* axis. Small adjustments in scope elevator, big wheel, short wire control, and tip deflection are preferable to forceful advancement. Contrast should be reserved for confirming ductal position or defining anatomy after access. A small amount of air or water can clear the field, but excessive insufflation can worsen duodenal loop instability. The assistant should keep the wire tip soft and mobile, avoiding a stiff wire protruding far beyond the catheter.

After several failed gentle attempts, the endoscopist should pause. The pause is not wasted time. It allows suctioning, reduction of duodenal distension, reassessment of papillary type, replacement of a suboptimal sphincterotome, or a change from long to short scope position. If the pancreatic duct has been entered, the team should decide whether this is an opportunity for pancreatic access-assisted cannulation or a warning to stop repeated trauma.

## Pancreatic access-assisted rescue techniques

Once difficulty is declared, the rescue pathway should depend on whether PD access is available. Repeated unintentional PD cannulation suggests that the catheter is aligned with the pancreatic rather than biliar*y* axis. In this setting, DGW cannulation, TPBS, or PD stent-assisted cannulation may redirect access toward the bile duct. These options, together with precut techniques, should not be interpreted as fixed successive steps. Selection should incorporate papillary morphology, the frequency and depth of PD access, ERCP indication, underlying anatomy, previous interventions, PEP and bleeding risk, and operator expertise. Direct comparative evidence exists for selected pairwise comparisons, but no trial validates a universal rescue sequence (1, 11–18).

DGW leaves a guidewire in the PD to straighten the common channel and occupy the pancreatic orifice while a second wire is advanced toward the bile duct. It is attractive because it does not require cutting, but it may increase pancreatic manipulation. A 2024 meta-analysis of 20 randomized controlled trials found that DGW increased cannulation success compared with persistent standard cannulation (11). Because DGW is a pancreatic guidewire-assisted technique, ESGE recommends attempting prophylactic PD stenting in patients undergoing pancreatic guidewire-assisted biliary cannulation (strong recommendation, moderate-quality evidence) (2). The 2025 WEO guideline conditionally suggests DGW or transpancreatic sphincterotomy after more than one deep unintentional PD cannulation, followed by PD stent placement after biliary cannulation (low-quality evidence) (1). ASGE also strongly recommends prophylactic PD stenting in high-risk patients when the PD has been repeatedly or deeply accessed (moderate-quality evidence) (4).

TPBS, also called transpancreatic septotomy, uses the PD position to incise the septum between the pancreatic and biliary ducts, opening the biliar*y* axis. For a small or flat papilla, TPBS is appropriate only when the guidewire has already obtained stable PD access. If useful PD access is absent, an expert needle-knife technique, including NKF when an adequate papillary roof is present or NKP when the orifice is visible, may be selected instead; morphology alone should not trigger intentional PD instrumentation. In a multicenter randomized trial, TPBS achieved a higher successful deep biliary cannulation rate than DGW, with similar PEP rates (12). Network meta-analyses have also suggested that TPBS may compare favorably with persistent standard techniques for cannulation success, although the certainty of evidence varies (13, 14). Recent commentaries, randomized data, comparative cohort data, and registry analyses have continued to refine the role of pancreatic guidewire-assisted fistulotomy, DGW, and TPBS in this setting (15–18). ESGE recommends considering TPBS in a small, difficult papilla when unintentional guidewire insertion into the PD occurs and suggests prophylactic PD stenting after TPBS (strong recommendation, moderate-quality evidence) (2). WEO similarly suggests DGW or transpancreatic sphincterotomy followed by PD stenting after repeated deep unintended PD cannulation (conditional recommendation, low-quality evidence) (1). Therefore, prophylactic PD stenting should generally be planned after TPBS when technically feasible.

## Precut access: timing, technique, and anatomy

Precut techniques are not a mark of failure; they are advanced access techniques that should be used before repeated trauma creates papillary edema. The main options are NKF, needle-knife papillotomy (NKP), and access over or beside a PD stent. The safety of precut depends heavily on operator experience, papillary anatomy, and timing. ESGE recommends NKF as the preferred precut technique (strong recommendation, moderate-quality evidence) and suggests that precut should be performed by endoscopists with high selective cannulation competence (weak recommendation, low-quality evidence) (2). ASGE guidance also supports early precut in difficult cannulation when expertise is available (3).

NKF starts above the papillary orifice on the intraduodenal biliary segment, creating a controlled fistula into the bile duct while avoiding the pancreatic orifice. It is particularly useful for a protruding or bulging papilla with a clearly visible roof and a bile duct dilated to the papilla. A 2024 systematic review comparing NKF with needle-knife papillotomy found broadly similar efficacy and safety, with the choice of technique depending on anatomy and expertise (19). Earlier meta-analyses of early precut have shown that early use does not increase overall adverse events and may reduce PEP compared with persistent standard cannulation attempts (20, 21). This supports a shift in mindset: the risk is often the prolonged failed attempt rather than the carefully performed early precut.

Needle-knife papillotomy starts at the orifice and cuts upward toward the biliar*y* axis. It may be useful when the orifice is visible but the bile duct cannot be deeply entered. Compared with NKF, it is closer to the pancreatic orifice and may carry more concern for pancreatic injury in some settings. PD stent-assisted precut can stabilize the papilla, identify the pancreatic axis, and protect pancreatic drainage while the incision is made toward the bile duct. This approach is reasonable when the PD has already been accessed easily. ESGE suggests placement of a PD stent before precutting when PD access is easy (weak recommendation, moderate-quality evidence) (2). By contrast, after NKF performed without PD instrumentation, routine intentional PD cannulation solely to place a prophylactic stent is not uniformly required by current guidelines; PD stenting may be considered when overall PEP risk is high and atraumatic PD access is easily achievable, consistent with the ASGE conditional recommendation for high-risk patients with readily achievable PD access (4).

## Device, electrosurgery, and procedural goal

Device selection should be deliberate. A pull-type sphincterotome with a 0.025 inch or 0.035 inch hydrophilic guidewire is the most common starting platform, but a straight cannula, tapered-tip catheter, rotatable sphincterotome, or different wire stiffness may be preferable when the papillar*y* axis is unusual. A 0.025 inch wire often provides a favorable balance between tip flexibility and torque transmission; a 0.018 inch wire can help in a very small or stenotic orifice, but device compatibility and wire control must be checked before the case. The accessory should be advanced only after the wire has clearly followed a ductal course; pushing the catheter against resistance increases papillary trauma and false passage risk.

Electrosurgical settings are relevant when sphincterotomy or precut is performed. Blended or endocut currents are commonly used because they balance cutting with hemostasis, whereas pure cut may increase immediate bleeding and excessive coagulation may deepen thermal injury. The practical objective is not a specific setting shared across all generators, but a short, controlled incision with frequent reassessment of bile staining, ductal exposure, and bleeding. Coagulopathy, thrombocytopenia, recent antithrombotic use, and portal hypertensive duodenopathy should lower the threshold for alternative drainage or staged therapy. If bleeding obscures the field during rescue access, the priority is visualization and hemostasis rather than further cutting.

The intervention planned after access also matters. For a small common bile duct stone, a limited sphincterotomy followed by balloon extraction may be sufficient. For distal malignant obstruction, deep access must be followed by reliable drainage, usually with a self-expandable metal stent if palliation is intended. For acute cholangitis, the goal is drainage rather than a perfect cholangiogram; excessive contrast injection into ducts that may not drain can worsen sepsis. These considerations should shape how long the endoscopist persists with transpapillary access before moving to an alternate drainage route.

## Special situations and altered anatomy

Periampullary diverticula, intradiverticular papillae, tumor-distorted papillae, and surgically altered anatomy require modification of the algorithm. In periampullary diverticulum, the challenge is often exposure and orientation. Cap-assisted visualization, gentle traction with a catheter or clip, PD stent-guided access, or NKF may be considered depending on papillary position. In malignant distal biliary obstruction, the papilla may be swollen, infiltrated, or hidden under a duodenal stricture. If duodenal access is unstable or the papilla is not reachable, early referral to endoscopic ultrasound-guided biliary drainage (EUS-BD) or percutaneous transhepatic biliary drainage (PTBD) may be safer than repeated transpapillary attempts.

In Roux-en-Y gastric bypass, hepaticojejunostomy, pancreaticoduodenectomy, or Billroth II anatomy, the main difficulty may be reaching the papilla or anastomosis rather than cannulating it. Enteroscopy-assisted ERCP, laparoscopy-assisted ERCP, endoscopic ultrasound-directed transgastric ERCP, EUS-BD, or interventional radiology approaches should be selected based on anatomy, indication, urgency, and local expertise (22, 23). These procedures should not be treated as interchangeable; each has different requirements for sedation, stent management, adverse event profile, and future surgical planning.

## Post-ERCP pancreatitis prevention as part of access strategy

PEP prevention must be embedded in the cannulation algorithm rather than added after difficult cannulation has already occurred. Rectal indomethacin or diclofenac 100 mg should be given before or during ERCP to patients without contraindications; ASGE and WEO provide strong recommendations based on moderate-quality evidence for rectal NSAIDs 1, 4. For practical decision-making, prophylactic PD stenting should be used after repeated or deep PD access, DGW, or TPBS when placement is technically feasible. After NKF that avoids PD instrumentation, routine intentional PD cannulation solely to place a prophylactic stent is not recommended; stenting should instead be individualized according to overall PEP risk and the ease of atraumatic PD access (1, 2, 4). When a prophylactic stent is indicated, we favor a short, soft plastic PD stent, 3-5 Fr in diameter (preferably 5 Fr), without an internal flange and 3–7 cm in length; a 2–3-cm stent may be used if the guidewire cannot be advanced beyond the pancreatic head (4). Spontaneous passage should be confirmed within 5–10 days, with endoscopic removal if the stent is retained (4, 6).

Hydration with lactated Ringer's solution can reduce PEP in selected patients and is suggested by ASGE for unselected patients (conditional recommendation, moderate-quality evidence), but it should be avoided or modified in patients at risk of fluid overload, including heart failure, advanced kidney disease, or decompensated cirrhosis (4). WEO also supports aggressive lactated Ringer's hydration when used as the principal pharmacologic-fluid prophylaxis in settings where rectal NSAIDs are unavailable (1). Sublingual nitrate has been discussed in some guidelines when NSAIDs or hydration are contraindicated, although it is not a substitute for careful technique (6). Antibiotic prophylaxis is not routinely required for all ERCPs, but it is appropriate when incomplete drainage is anticipated, in severely immunocompromised patients, or during selected cholangioscopy and drainage scenarios (6).

Local postprocedural cryoprevention has emerged as a potential adjunct. In a 2025 multicenter, single-blind randomized trial of 877 adults with a native papilla, irrigating the papilla with 250 mL of ice water in five 50-mL aliquots immediately after ERCP reduced PEP from 6.8% to 3.2% (*P* = 0.02), with no adverse events attributed to cooling (24). However, the trial did not use rectal NSAIDs, had no sham or room-temperature irrigation group, and did not establish the incremental benefit of cooling when guideline-based NSAIDs or prophylactic PD stenting are used. We therefore consider cryoprevention an optional, low-cost adjunct, particularly where rectal NSAIDs are unavailable or contraindicated, but not a replacement for established prophylaxis pending further external validation.

## Bailout after failed biliary cannulation

When biliary access fails despite standard and advanced techniques, the safest next step is often to stop. Subsequent management should be individualized according to the underlying indication, urgency and clinical stability, native or altered anatomy, level and cause of obstruction, prior interventions, future surgical planning, and the availability of advanced endoscopic and interventional radiology expertise. In stable benign disease with native anatomy when urgent drainage is not required, repeat ERCP in a high-volume center after an interval of at least 48 h is reasonable; WEO makes this a conditional recommendation based on very low-quality evidence (1). If urgent drainage is required or repeat ERCP is unsuitable, EUS-guided rendezvous can preserve transpapillary access and is favored in suspected benign disease when technically feasible; WEO strongly recommends EUS-guided rendezvous when expertise is available (moderate-quality evidence), and ASGE lists rendezvous as the preferred EUS approach over transmural drainage in suspected benign disease (1, 23). For malignant biliary obstruction, randomized-trial meta-analysis found similar technical and clinical success for EUS-BD and ERCP-guided drainage as initial drainage, with lower reintervention and PEP after EUS-BD (28). In distal malignant biliary obstruction after failed ERCP, ESGE recommends EUS-BD over PTBD when local expertise is available (strong recommendation, moderate-quality evidence), while ASGE conditionally suggests EUS-BD over PTBD after failed ERCP (low-quality evidence) (22, 23). PTBD remains important when EUS expertise is unavailable, the patient is hemodynamically unstable or cannot tolerate advanced endoscopy or anesthesia, or anatomy and obstruction characteristics make an EUS approach unsuitable. Hilar or proximal obstruction, altered anatomy, and potentially resectable malignancy require particularly careful multidisciplinary planning.

Failed cannulation should trigger a structured handoff, not an isolated procedural decision. The report should document the indication, papillary morphology, duration of attempts, number of pancreatic duct passages, whether contrast entered the pancreatic duct, rescue techniques used, PD stent placement, NSAID administration, and the reason for stopping. This information helps the next endoscopist decide whether repeat ERCP, EUS-rendezvous, EUS-BD, or interventional radiology drainage is most appropriate. In acute cholangitis, antibiotics and source control should be coordinated with surgery and interventional radiology; in potentially resectable malignancy, drainage route should be discussed with oncology and surgery because transmural stents may affect future operative planning.

A practical algorithm is shown in Figure 2. The key decision nodes are: declare difficulty early; determine whether PD access is present; select DGW, TPBS, PD stent-assisted access, or an expert precut according to anatomy and clinical context; protect the PD when it has been repeatedly or deeply instrumented; and move to expert rescue or alternative drainage after failed access. Figure 3 summarizes a morphology-based rescue matrix. Both figures should be interpreted as cognitive aids rather than rigid rules or a mandatory priority ranking. Guideline recommendation strength and evidence quality for the major decision nodes are summarized in Table 2. The precise sequence among rescue techniques is not directly validated by high-quality comparative trials and should be influenced by the indication, papillary morphology, ductal dilation, PD access, coagulopathy and antithrombotic status, cholangitis, patient stability, previous interventions, available expertise, and whether definitive therapy can be completed after access.

**Table 2:** Practical evidence-informed algorithm for difficult biliary cannulation.

Decision point	Recommended action	Evidence basis and strength
Before cannulation	Give rectal NSAID before or during ERCP unless contraindicated; assess PEP and bleeding risk and plan hydration if suitable.	ASGE and WEO: strong recommendation, moderate-quality evidence for rectal NSAIDs (1, 4). Hydration: ASGE conditional recommendation, moderate-quality evidence (4).
First-line cannulation	Use wire-guided cannulation with minimal contrast and controlled papillary contact.	WEO and ESGE: strong recommendation, moderate-quality evidence; ASGE: conditional recommendation, moderate-quality evidence (1, 2, 4, 5).
Difficulty threshold reached	Stop persistent standard attempts and reassess papillary morphology, PD access, patient risk, and operator options.	ESGE 5-5-1 definition: weak recommendation, low-quality evidence (2). The exact switch point within these thresholds remains clinical judgment.
Repeated or deep PD access	Choose DGW or TPBS according to papillary anatomy and expertise; use TPBS only when PD access is already established; place a prophylactic PD stent when feasible.	WEO, ESGE, and ASGE support prophylactic PD stenting after pancreatic guidewire-assisted or repeated/deep PD access (1, 2, 4).
Favorable precut anatomy without useful PD access	Use early expert precut, preferably NKF when appropriate; do not force a pancreatic access-assisted technique solely to follow a sequence. For a small/flat papilla without useful PD access, consider expert NKF when an adequate roof is present or NKP when the orifice is visible.	ESGE prefers NKF for precut when anatomy is suitable; WEO supports anatomy- and expertise-based advanced access when no unintended PD cannulation has occurred (1, 2).
Prophylactic PD stent selected	Use a short, soft plastic 3–5-Fr stent, preferably 5 Fr, without an internal flange and 3–7 cm long; use 2–3 cm if the wire cannot pass beyond the pancreatic head. Confirm passage within 5–10 days and remove if retained.	ASGE practical stent specification and ESGE follow-up principles (4, 6).
After ERCP: optional cryoprevention	Consider 250 mL of ice water directed at the papilla in five 50-mL aliquots as an adjunct, especially when NSAIDs cannot be used; do not substitute it for established prophylaxis.	One 2025 multicenter RCT reduced PEP from 6.8% to 3.2%, but NSAIDs were not used and incremental benefit with standard prophylaxis is unknown (24).
Failed access	Stop traumatic attempts. If stable and drainage can wait, arrange repeat expert ERCP after at least 48 h; if urgent or persistent, select EUS-RV, EUS-BD, or PTBD according to disease, anatomy, stability, future surgery, and expertise.	WEO supports interval expert ERCP or rendezvous rescue; ESGE and ASGE support EUS-guided drainage over PTBD in selected settings with expertise (1, 22, 23).

## Training, quality metrics, and future directions

Quality in biliary cannulation should be measured by safe access, not merely by eventual access. Useful unit-level metrics include native papilla cannulation success, rate of difficult cannulation, early rescue technique use, PEP rate stratified by risk, PD stent placement after repeated pancreatic duct access, failed ERCP referral pathways, and adverse events after precut. Simulation, video-based review, and structured mentorship can help trainees recognize papillary orientation and the moment when persistence becomes harmful.

Future research should compare anatomy-driven rescue algorithms rather than isolated techniques. Recent guidelines have highlighted the need for better evidence on optimal sequencing, prophylactic stenting thresholds, electrosurgical settings, and rescue after failed cannulation (1). In particular, head-to-head trials are needed to determine whether specific papillary morphologies or patterns of PD access should preferentially direct DGW, TPBS, PD stent-assisted precut, or NKF. Artificial intelligence may eventually support papillary classification, scope positioning, and wire trajectory feedback, but these tools remain investigational and must be validated against patient-centered outcomes such as PEP, successful drainage, reintervention, and hospitalization. Practical reviews and expert commentaries have similarly emphasized that algorithms should remain flexible and should prioritize early expert rescue over persistence with injurious attempts (25–27). Until then, the most important innovation is disciplined decision-making: a team that declares difficulty early, chooses rescue deliberately, and stops before preventable injury accumulates.

## Conclusion

Difficult biliary cannulation is a predictable and manageable phase of ERCP when it is recognized early and approached systematically. Standard wire-guided cannulation should be concise and atraumatic. Once objective difficulty criteria are met, rescue should be individualized according to papillary morphology, PD access, indication, anatomy, patient risk, and operator expertise rather than imposed as a fixed sequence. DGW, TPBS, or PD stent-assisted techniques are reasonable when the PD is repeatedly entered; early expert NKF or papillotomy may be favored when papillary anatomy supports precut; and repeat expert ERCP, EUS-guided rescue, or PTBD should be selected according to disease setting, urgency, stability, and local expertise when transpapillary access fails or is unsafe. PEP prevention is inseparable from cannulation strategy and should begin before or during ERCP, with rectal NSAIDs, scenario-specific prophylactic PD stenting, and appropriate hydration. Local papillary cryoprevention may be considered as an emerging adjunct, but it should not replace guideline-supported measures until its incremental benefit with standard prophylaxis is established. The practical endpoint is not simply reaching the bile duct, but achieving durable biliary drainage while avoiding preventable pancreatic injury, bleeding, perforation, cholangitis, and unplanned reintervention. A flexible, evidence-informed algorithm that clearly distinguishes guideline-supported nodes from areas of expert-practice synthesis can improve transparency and support safer clinical decision-making.

## References

[1] Crinò SF Aabakken L Bapaye A Chan S Kida M Law R. World endoscopy organization guidelines on endoscopic retrograde cholangiopancreatography biliary cannulation and sphincterotomy techniques. Dig Endosc. (2025) 37(10):1029–53. 10.1111/den.15060 40518920

[2] Testoni P Mariani A Aabakken L Arvanitakis M Bories E Costamagna G. Papillary cannulation and sphincterotomy techniques at ERCP: european society of gastrointestinal endoscopy (ESGE) clinical guideline. Endoscopy. (2016) 48(7):657–83. 10.1055/s-0042-108641 27299638

[3] Chandrasekhara V Khashab MA Muthusamy VR Acosta RD Agrawal D Bruining DH. Adverse events associated with ERCP. Gastrointest Endosc. (2017) 85(1):32–47. 10.1016/j.gie.2016.06.051 27546389

[4] Buxbaum JL Freeman M Amateau SK Chalhoub JM Coelho-Prabhu N Desai M. American Society for gastrointestinal endoscopy guideline on post-ERCP pancreatitis prevention strategies: summary and recommendations. Gastrointest Endosc. (2023) 97(2):153–62. 10.1016/j.gie.2022.10.005 36517310

[5] Tse F Liu J Yuan Y Moayyedi P Leontiadis GI. Guidewire-assisted cannulation of the common bile duct for the prevention of post-endoscopic retrograde cholangiopancreatography (ERCP) pancreatitis. Cochrane Database Syst Rev. (2022) 3:CD009662. 10.1002/14651858.CD009662.pub3 35349163 PMC8963249

[6] Dumonceau J-M Kapral C Aabakken L Papanikolaou IS Tringali A Vanbiervliet G. ERCP-related adverse events: european society of gastrointestinal endoscopy (ESGE) guideline. Endoscopy. (2020) 52(2):127–49. 10.1055/a-1075-4080 31863440

[7] Haraldsson E Lundell L Swahn F Enochsson L Löhr JM Arnelo U. Endoscopic classification of the papilla of vater. Results of an inter- and intraobserver agreement study. United Eur Gastroenterol J. (2017) 5(4):504–10. 10.1177/2050640616674837 PMC5446150 28588881

[8] Tari E Gagyi EB Rancz A Veres DS Váncsa S Hegyi PJ. Morphology of the papilla can predict procedural safety and efficacy of ERCP-a systematic review and meta-analysis. Sci Rep. (2024) 14:7341. 10.1038/s41598-024-57758-9 38538734 PMC10973369

[9] Han S Zhang J Durkalski-Mauldin V Foster LD Serrano J Coté GA. Impact of difficult biliary cannulation on post-ERCP pancreatitis: secondary analysis of the stent versus indomethacin trial dataset. Gastrointest Endosc. (2025) 101(3):617–28. 10.1016/j.gie.2024.10.003 39389431 PMC11875935

[10] Chen PH Tung CF Peng YC Yeh HZ Chang CS Chen CC. Duodenal major papilla morphology can affect biliary cannulation and complications during ERCP, an observational study. BMC Gastroenterol. (2020) 20:310. 10.1186/s12876-020-01455-0 32988368 PMC7520951

[11] Wang L Wang L Huang N Li T Shi X Zhu Q. Comparison between different advanced cannulation techniques for difficult biliary cannulation: a systematic review with a meta-analysis. Front Med (Lausanne). (2024) 11:1344644. 10.3389/fmed.2024.1344644 38716417 PMC11074372

[12] Kylänpää L Koskensalo V Saarela A Ejstrud P Udd M Lindström O. Transpancreatic biliary sphincterotomy versus double guidewire in difficult biliary cannulation: a randomized controlled trial. Endoscopy. (2021) 53(10):1011–9. 10.1055/a-1327-2025 33440441

[13] Facciorusso A Ramai D Gkolfakis P Khan SR Papanikolaou IS Triantafyllou K. Comparative efficacy of different methods for difficult biliary cannulation in ERCP: systematic review and network meta-analysis. Gastrointest Endosc. (2022) 95(1):60–71.e12. 10.1016/j.gie.2021.09.010 34543649

[14] Yang QI Yao W Li Q Yao W Kong L Xu R. Efficacy of different methods for difficult biliary cannulation in ERCP: systematic review and network meta-analysis. J Surg Concepts Pract. (2023) 28(6):551–8. 10.16139/j.1007-9610.2023.06.010

[15] Nabi Z Reddy DN. Navigating difficult biliary cannulation: is pancreatic guidewire-assisted fistulotomy the preferred route? Indian J Gastroenterol. (2025) 44(6):742–5. 10.1007/s12664-025-01797-1 40488986

[16] Sadeghi A Arabpour E Rastegar R Hosseinzadeh E Tape PMK Zali MR. Pancreatic guidewire-assisted fistulotomy versus transpancreatic biliary sphincterotomy in difficult biliary cannulation with unintentional pancreatic duct cannulation: a randomized clinical trial. Indian J Gastroenterol. (2025) 44(6):862–71. 10.1007/s12664-025-01774-8 40448903

[17] Bao J Wang S Bai B Huang Q Zhang L Chen X. Comparison of preferred double guidewire technique and transpancreatic sphincterotomy for difficult biliary cannulation. Sci Rep. (2025) 15:41314. 10.1038/s41598-025-25120-2 41271923 PMC12639170

[18] Pécsi D Farkas N Gódi S Hegyi P Szentesi A Altorjay I. Transpancreatic sphincterotomy is a safe and effective pancreatic guidewire-assisted cannulation method: real-world data analysis of the Hungarian ERCP registry. J Clin Med. (2025) 14(19):7118. 10.3390/jcm14197118 PMC12525139 41096200

[19] Alsakarneh S Brotherton T Jaber F Madi MY Numan L Ahmed M. Needle-Knife fistulotomy versus needle-knife papillotomy in difficult biliary cannulation: a systematic review and meta-analysis. Gastroenterol Res. (2024) 17(3):101–8. 10.14740/gr1726 PMC11236341 38993545

[20] Chen J Wan JH Wu DY Shu WQ Xia L Lu NH. Assessing quality of precut sphincterotomy in patients with difficult biliary access: an updated meta-analysis of randomized controlled trials. J Clin Gastroenterol. (2018) 52(7):573–8. 10.1097/MCG.0000000000001077 29912752

[21] Tang Z Yang Y Yang Z Meng W Li X. Early precut sphincterotomy does not increase the risk of adverse events for patients with difficult biliary access: a systematic review of randomized clinical trials with meta-analysis and trial sequential analysis. Medicine (Baltimore). (2018) 97(36):e12213. 10.1097/MD.0000000000012213 30200135 PMC6133433

[22] van Der Merwe SW Van Wanrooij RLJ Bronswijk M Everett S Lakhtakia S Rimbas M. Therapeutic endoscopic ultrasound: european society of gastrointestinal endoscopy (ESGE) guideline. Endoscopy. (2022) 54(2):185–205. 10.1055/a-1717-1391 34937098

[23] Pawa S Marya NB Thiruvengadam NR Ngamruengphong S Baron TH Bun Teoh AY. American Society for gastrointestinal endoscopy guideline on the role of therapeutic EUS in the management of biliary tract disorders: summary and recommendations. Gastrointest Endosc. (2024) 100(6):967–79. 10.1016/j.gie.2024.03.027 39078360

[24] Azuma S Kobayashi Y Harada R Yane K Sawada K Tsujimoto A. Local postprocedural cryoprevention significantly reduces the incidence of post-ERCP pancreatitis: a multicenter randomized controlled trial. Am J Gastroenterol. (2025) 121:1224–32. 10.14309/ajg.0000000000003644 40638082

[25] Berry R Han JY Tabibian JH. Difficult biliary cannulation: historical perspective, practical updates, and guide for the endoscopist. World J Gastrointest Endosc. (2019) 11(1):5–21. 10.4253/wjge.v11.i1.5 30705728 PMC6354112

[26] Weismüller TJ Trivedi PJ Bergquist A Imam M Lenzen H Ponsioen CY. How to improve cannulation rates during endoscopic retrograde cholangiopancreatography. Gastroenterology. (2017) 152(6):1275–9. 10.1053/j.gastro.2017.02.038 28366733

[27] Angsuwatcharakon P Piyachaturawat P Pantongrag-Brown L Rerknimitr R. Difficult biliary cannulation: the resilience of old algorithms and the promise of safer rescue. Gastrointest Endosc. (2022) 95(1):72–4. 10.1016/j.gie.2021.10.01034237329

[28] Barbosa EC do Espiroto Santo PA Baraldo S Nau AL Meine GC. EUS- versus ERCP-guided biliary drainage for malignant biliary obstruction: a systematic review and meta-analysis of randomized controlled trials. Gastrointest Endosc. (2024) 100(3):395–405.e8. 10.1016/j.gie.2024.04.019 38648989

